# Sexual Dysfunction Among Young Adults in Sweden—A Population-Based Observational Study

**DOI:** 10.1016/j.esxm.2020.08.010

**Published:** 2020-09-30

**Authors:** Lisa Ljungman, Claudia Lampic, Lena Wettergren

**Affiliations:** 1 Department of Women's and Children's Health, Uppsala University, Uppsala, Sweden; 2 Department of Women's and Children's Health, Karolinska Institutet, Stockholm, Sweden; 3 Department of Public Health and Caring Sciences, Uppsala University, Uppsala, Sweden

**Keywords:** Sexual Dysfunction, Young Adults, Body Image, Depression, Anxiety, Sexual Activity

## Abstract

**Introduction:**

There is a lack of studies using validated instruments to investigate prevalence and predictors of sexual dysfunction among young adults.

**Aim:**

This population-based observational study aimed to determine the prevalence and predictors of sexual dysfunction in young adults in Sweden and to compare sexual function in women and men.

**Methods:**

A random sample of the general population aged 19-40 years, identified via the Swedish population registry, was approached with a postal survey. A total of 819 individuals participated, 493 women (51% response) and 326 men (34% response). Predictors of sexual dysfunction were identified by multivariable logistic binary regression analyses.

**Main outcome measure:**

Sexual function and satisfaction were assessed using the Patient-Reported Outcomes Measurement Information System Sexual Function and Satisfaction measure, version 2.0.

**Results:**

Among the women, 53% reported at least one sexual dysfunction; the corresponding figure for men was 31%. The most common sexual dysfunction in women was low sexual interest (reported by 32%), whereas low satisfaction with sex life was the most common dysfunction in men (reported by 17%). Men reported a higher level of sexual interest and orgasm ability than women, whereas women reported a higher level of orgasm pleasure than men. Regression models showed that in both women and men, having a partner was related to lower risk of dysfunction in the domains satisfaction with sex life and orgasm pleasure. Having children was related to low interest in sex in women, whereas it was related to dissatisfaction with sex life in men. Being born outside of Sweden predicted sexual dysfunction in both women and men, as did experiencing symptoms of anxiety and depression.

**Conclusion:**

Sexual dysfunction is common in young adults, particularly in women. Risk factors of sexual dysfunction include not having a partner, having children, being an immigrant, and reporting symptoms of anxiety and depression.

**Ljungman L, Lampic C, Wettergren L, et al. Sexual Dysfunction Among Young Adults in Sweden—A Population-Based Observational Study. Sex Med 2020;8:631–642.**

## Background

Sexual dysfunction is a term used to describe various sexual problems including low desire or interest, diminished arousal, orgasmic difficulties, and dyspareunia.^1^^,^^2^ Impaired sexual function is associated with decreased mental health and low overall life satisfaction in both women and men.^3^ In the previous literature, common risk factors associated with sexual dysfunction for women and men include poor general health status, the presence of chronic illnesses such as diabetes mellitus, cardiovascular or genitourinary disease, psychiatric/psychological disorders, and sociodemographic factors.^1^ There is however a lack of studies investigating the prevalence and predictors of sexual dysfunction in young adults (younger than 40 years) of the general population, hampering firm conclusions with regard to both the extent of problems in this age group and of risk factors associated with them.

### Sexual Function in Women

Sexual activity is considered to be part of the concept sexual function, and most young women are sexually active. In a Dutch population-based study, about 80% of women of ages 20-40 years reported having engaged in any sexual activity during the past 30 days.^4^ Similarly, in a large U.K. study, 75% of women of ages 25-34 years report having had vaginal sex during the past 30 days, and 44% report having masturbated.^5^ Previous results suggest that the prevalence of women of all ages who report at least one sexual dysfunction is approximately 40–50%.^6^ It has also been reported that sexual problems in women have a nonlinear relationship with age, and that distressing sexual problems are more common in middle-aged women than in younger^7^, ^8^, ^9^, ^10^, ^11^, ^12^, ^13^, ^14^, ^15^, ^16^, ^17^, ^18^, ^19^, ^20^, ^21^, ^22^, ^23^, ^24^, ^25^, ^26^, ^27^, ^28^, ^29^, ^30^, ^31^, ^32^ or older women (>65 years).^2^ A population-based study from Australia has also shown that the degree of sexual problems varies with age where younger women are more likely to report pain during intercourse and older women are more likely to report lack of interest in sex, inability to reach orgasm, and vaginal dryness.^33^ Hayes et al^34^ concluded a consistent pattern in previous studies showing that the most common sexual dysfunction in women is lack of sexual desire, followed by orgasm difficulties, arousal difficulties, and pain associated with sexual activity. In a large U.S. study including more than 30,000 women, among those younger than 40 years, about a third reported problems related to sexual desire and/or orgasm.^2^ A longitudinal study from Australia (n = 2252, age 20-64 years) showed similar results by reporting “lacking interest in having sex” in 26% of the women and “taking too long to orgasm” in 11% of the women.^35^ The prevalence of sexual dysfunction in women has been reported to vary between countries. In a study including a large number of women (older than 40 years) in 29 countries, a higher prevalence of problems was reported in Southeast Asia followed by East Asia and the Middle East, whereas the lowest levels of dysfunction were reported in Northern Europe.^36^

In a review by Shahhosseini et al,^37^ mental health was suggested to be the strongest influencing factor on female sexual satisfaction. Depression and anxiety have additionally been strongly associated to problems with sexual desire, arousal, and orgasm in women.^2^ Having a negative body image is another factor reported to predict sexual dysfunction in young women, mainly dysfunction related to desire and arousal.^38^ It has also been reported that infertility-related distress is associated with female sexual dysfunction.^39^ Furthermore, higher education and having a partner have been associated with higher satisfaction with sex life,^37^^,^^40^^,^^41^ whereas women living without a partner have reported more orgasm difficulties and physical pain during intercourse.^35^ With regard to physical illnesses, increased levels of sexual problems have been reported after a range of medical conditions, for example, cancer^42^ and rheumatoid arthritis.^43^ Finally, a low level of physical activity and the use of antidepressants have been associated to sexual problems in women.^1^

### Sexual Function in Men

Among young men (age 25-34 years), 75% report having had vaginal sex during the past 30 days, and 78% report having masturbated.^5^ With regard to sexual dysfunctions, the most widely studied sexual problem in men is erectile dysfunction (ED). Prevalence rates of ED however vary widely between studies, which has been associated to a variance in methodologies in previous research.^6^ It has also been reported that the prevalence varies between countries, with men in the United States reporting higher rates of ED than men in Brazil, Mexico, and European countries.^7^ In a review of the literature, McCabe et al^44^ reported that 1–10% of men younger than 40 years report ED and that the prevalence increases significantly after 40 years.^6^ With regard to dysfunction related to decreased desire or interest, prevalence rates have been reported to be somewhat higher; 20–22% in men aged 20-39 years.^33^ Difficulties to achieve an orgasm, or delayed orgasm, has been reported by 8% of men aged 18-40 years in the United States.^8^ In a global study of men older than 40 years, it was reported that the prevalence of orgasm dysfunction was 5–8% in most areas of the world, except in East and Southeast Asia where the prevalence was 10–15%.^36^ However, there are few studies reporting orgasm problems in young men, and a clarification of the definition of male orgasmic disorders has been called for to determine prevalence rates.^9^ In a large study from Australia, it was reported that the sexual problems with the highest incident in men (age 20-64 years) were “lacking interest in having sex” (11%) and “reaching orgasm too quickly” (7%).^10^ If unspecified as to type of sexual problems, it has been reported that 31% of men up to the age of 59 years report any sexual dysfunction.^11^ Still, figures with regard to prevalence of sexual problems specifically in younger men are lacking.

Several risk factors for sexual dysfunction in men have been identified. Nguyen et al^12^ concluded in a review of the literature that anxiety and depression are associated to ED in young men and furthermore that there is a reciprocal relationship between these factors and ED. It is also well-known that ED increases with age^7^^,^^13^ and has been associated to various physical illnesses including, for example, cardiovascular, endocrine, and neurological diseases.^14^ Additional risk factors for ED include smoking, high alcohol consumption, and low physical activity level.^1^ Although sexual satisfaction has been reported to be higher in young adults who have a partner relationship than in unpartnered individuals, it seems to decrease over the course of a partner relationship.^41^ Interestingly, in a study using longitudinal data, it has been shown that men (30–39 years) who lived apart from their female partner were significantly less likely to report feeling anxious about their ability to perform sexually compared with those living with their partner.^10^ There are very few studies examining predictors of orgasm problems in men, but it has been suggested that general anxiety and relationship difficulties increase the risk of this type of sexual dysfunction.^9^

Taken together, knowledge about the prevalence of sexual activities and different types of sexual dysfunctions in young women and men is sparse. In addition, the mechanisms involved in sexual dysfunction in this age group are not fully understood, and the relative impact of psychological, sociodemographic, and physical factors is yet to be determined. Sexual dysfunction in young adulthood is associated with suffering and may also interfere with important life goals such as building intimate relationships and having children. Increased knowledge about risk factors is needed to enable early detection and the possibility to offer adequate treatment.

### Aim

This study aimed to determine the prevalence and predictors of self-reported sexual dysfunction in the general population of young adults (age 19-40 years) in Sweden and to compare sexual function in women and men.

## Methods

### Design and Context

The present study had a population-based cross-sectional design. The study was part of a larger research program investigating sexual function and fertility-related concerns in young adults diagnosed with cancer (Fex-Can) (see the study protocol by Lampic et al)^15^ The present study was conducted to collect comparison data for young adults of the general population in Sweden. Individuals who had been treated for cancer previously were excluded to provide figures for a cancer-free population.

### Participants and Procedure

Participants were identified using the Swedish population registry, which includes all persons registered as residents in Sweden. The Swedish population registry provided a random sample of 2000 individuals (1,000 women and 1,000 men) in the age group 19-40 years. With an expected response rate of approximately 50%, we estimated the sample size to be sufficient for the statistical analyses, see the following sections. The potential participants were approached via post including a letter with information about the study, written informed consent, a prepaid envelope for survey return, and the survey with the self-administered measures. Written informed consent was thereby given by all participants in the study. It was optional to complete the survey via paper or web; login information and the link to the web-survey were provided to participants. 2 reminders were sent to nonresponders. Individuals without address information were excluded. In addition, individuals who reported having cognitive impairments hindering them from participating in the study and individuals who reported having been treated for cancer were excluded. 2 cinema tickets were provided as incentives for study participation to participants completing the survey.

Ethical approval of the study procedures has been obtained from the Regional Ethical Review Board in Stockholm (Dnr: 2013/1746-31/4; 2014/2244-32; 2017/916-32). Data were collected from April to June, 2018.

### Measurements

#### Main Outcome

##### Sexual Function

Sexual function and satisfaction were assessed using the Patient-Reported Outcomes Measurement Information System Sexual Function and Satisfaction measure, version 2.0 (PROMIS SexFS).^16^ Development of the instrument's domain structure was based on literature review and expert opinion, which were subsequently related to qualitative and quantitative evidence. The PROMIS SexFS has shown adequate content and construct validity and reliability^16^ and is customizable as it allows selection of relevant items and domains. With the exception of the domain interest in sexual activity, items are completed by individuals who have had sexual activity of any kind during the past 30 days. Sexual activity is defined as sex with a partner and/or solo sex, thus including, for example, masturbation, oral sex, and sexual intercourse. For the present study, the following domains of the SexFS were selected for women and men: “Satisfaction with sex life”, “Interest in sexual activity”, “Orgasm–Pleasure”, “Orgasm–Ability.” For women only, the domains “Vaginal lubrication”, “Vaginal discomfort”, “Vulvar discomfort–Labia,” and “Vulvar discomfort–Clitoral” were selected. In addition, for men, the domain “Erectile function” was selected. Item response theory is used to estimate the respondent's item responses and transform these to a t-score metric (mean = 50, SD = 10). The mean of 50 corresponds to the mean of the population of U.S. adults who have been sexually active in the past 30 days.^16^ One SD (10 points on the t-scale) less than or more than 50 (depending on the direction of the scale that indicates dysfunction) is considered indicative of dysfunction in that domain. The definition of sexual dysfunction is thus based on individual scores related to the mean scores of the U.S. norm data.^16^ Lower scores indicate more problems in the following domains: “Satisfaction with sex life,” “Interest in sexual activity,” “Orgasm–Pleasure,” “Orgasm–Ability,” “Vaginal lubrication,” and “Erectile function.” In the following domains, higher scores indicate more problems: “Vaginal discomfort,” “Vulvar discomfort–Labia,” and “Vulvar discomfort–Clitoral.” In addition, items regarding sexual activity and an item concerning reasons not to having had sex with a partner during the past 30 days were included. The Swedish version of the PROMIS SexFS has been translated according to the principles of the FACITrans and PROMIS.^17^ Missing data were handled according to the established PROMIS methodology for the SexFS.^16^ Cronbach's alpha for the selected domains in the present study exceeded 0.70 for all domains except Erectile function (Cronbach's alpha 0.52).

**Table 1:** Participant characteristics reported for women and men (age 19-40 years) and figures for the Swedish population

Characteristics	Women		Men	
	Participants† (n = 493) n (%)	Swedish population‡^,^§%	Participants† (n = 326) n (%)	Swedish population‡^,^§%
Birth country				
Sweden	422 (86)	75	271 (83)	74
EU country	36 (7)		25 (8)	
Non-EU country	35 (7)		29 (9)	
Education				
University	283 (57)∗	54	150 (46)	40
Upper secondary school	179 (36)∗	34	153 (47)	45
Elementary school	18 (4)	9	13 (4)	12
Other	10 (2)		8 (3)	
Occupation				
Full-time employment	288 (58)∗	69ǁ	230 (71)	76ǁ
Part-time employment	77 (16)∗		23 (7)	
Student	75 (15)	23	52 (16)	17
Unemployed	14 (3)	8	6 (2)	6
Sick leave	19 (4)		6 (2)	
Other	19 (4)		6 (2)	
Partnered				
Yes	394 (81)∗∗	79	231 (72)	65
Have children				
Yes	230 (47)∗		127 (39)	
Wish for (additional) children				
Yes	281 (57)		187 (58)	
Uncertain	110 (22)		79 (25)	
No	99 (20)		56 (17)	
Sexual orientation				
Heterosexual	454 (93)		307 (95)	
Homosexual	6 (1)		7 (2)	
Bisexual	26 (5)∗		7 (2)	
Other	3 (1)		3 (1)	
Do not want to declare	1 (0.2)		0	

† The chi-square test for comparison between men and women of study population. ∗*P* < .05; ∗∗*P* < .01.

‡ Figures for the Swedish population are drawn from Statistics Sweden and reported where available.^45^

§ Comparison data age groups: Birth country 20-39 years; Education 25-44 years; occupation 20-39 years; partnered 20-39 years.

ǁ The figures reported at full-time also include part-time occupation as these are presented together by Statistics Sweden.

**Table 2:** Descriptive statistics for PROMIS SexFS, RCAC, BIS, EORTC QLQ-C30, and HADS and differences between women and men in PROMIS SexFS

PROMIS SexFS∗ domains	Women		Men				
	Mean (SD)	Score indicating dysfunction n (%)†	Mean (SD)	Score indicating dysfunction n (%)†	Chi-square test	t-Test	Cronbach's alpha Women/men
Vaginal lubrication	52.4 (7.5)	30 (7)	N/A	N/A	N/A	N/A	0.86
Vaginal discomfort	49.3 (7.6)	35 (8)	N/A	N/A	N/A	N/A	0.81
Vulvar discomfort clitoral	51.1 (6.8)	66 (16)	N/A	N/A	N/A	N/A	N/A
Vulvar discomfort labia	50.6 (6.7)	80 (19)	N/A	N/A	N/A	N/A	N/A
Satisfaction with sex life	51.3 (8.2)	51 (12)	50.4 (8.8)	52 (17)	4.6‖	1.3	0.87/0.87
Sexual interest	46.3 (10.9)	156 (32)	52.4 (8.9)	32 (10)	52.0#	-8.7#	0.83/0.75
Erectile function	N/A	N/A	53.2 (5.6)	11 (4)	N/A	N/A	0.52
Orgasm ability	48.5 (10.4)	117 (28)	55.0 (6.1)	16 (5)	59.1#	-10.4#	N/A
Orgasm pleasure	51.0 (7.9)	21 (5)	49.6 (7.7)	17 (6)	0.1	2.3‖	N/A
At least one sexual dysfunction‡	N/A	263 (53)	N/A	98 (31)	41.4#		
At least 2 sexual dysfunctions‡	N/A	77 (16)	N/A	24 (7)	11.9¶		
	Mean (SD)	Score indicating distress (%)§	Mean (SD)	Score indicating distress (%)§			
BIS	5.2 (3.6)	N/A	3.2 (2.7)	N/A			0.85
HADS-T	12.0 (7.4)	N/A	9.5 (5.9)	N/A			0.87
HADS-A	7.4 (4.5)	N/A	5.5 (3.8)	N/A			0.84
HADS-D	4.5 (3.6)	N/A	4.0 (2.9)	N/A			0.77
RCAC							
Fertility potential	2.7 (1.0)	42 (9)	2.1 (0.9)	5 (2)			0.77
Acceptance	2.8 (1.1)	65 (13)	2.6 (1.1)	29 (9)			0.80
Becoming pregnant	2.7 (0.9)	33 (7)	2.3 (0.8)	2 (1)			0.60
QLQ-C30	80.0 (14.0)	N/A	85.1 (9.9)	N/A			0.77

PROMIS SexFS = Patient-Reported Outcomes Measurement Information System Sexual Function and Satisfaction measure, version 2.0; RCAC = Reproductive Concerns After Cancer; BIS = Body Image Scale; HADS = Hospital Anxiety and Depression Scale.

∗ Answered by individuals who have had sexual activity (with or without partner) during past 30 days.

† PROMIS SexFS cutoff = 1 SD from the T-score mean of the norm population.^49^

‡ Summary of dysfunctions excluding Vaginal discomfort, Vulvar discomfort Clitoral, and Vulvar discomfort Labia to enable comparisons between women and men.

§ RCAC cutoff = mean > 4 per dimension.

‖ *P* < .05

¶ *P* < .01

# *P* < 0,01

#### Predictor Variables

##### Anxiety and Depression

Symptoms of anxiety and depression were measured using the Hospital Anxiety and Depression Scale (HADS).^18^ The HADS includes 14 items, 7 of which assess symptoms of depression and 7 assess symptoms of anxiety. Each item has a Likert response scale, and scores are constructed by summation of item responses with higher scores indicating increasing symptom burden. The scale can be summated in terms of the subscales separately (HADS-A, HADS-D) or can be used as a total scale.^19^ In the present study, we use the HADS total scale (HADS-T) as predictor in the regression models, and missing items were handled as missing. The internal consistency of the HADS has been reported to be satisfactory and the concurrent validity good.^20^ The Swedish version of the HADS has been used in previous studies and proven to be a valid indicator of possible depression and anxiety.^21^ Cronbach's alpha in the present study was 0.87 for HADS-T, and 0.84 for HADS-A and 0.77 for HADS-D.

##### Body Image

Body image was measured using the Body Image Scale (BIS). The original version of the BIS assesses body image discomfort in general (5 items) and is associated with cancer and cancer treatment (5 items).^22^ Responses are given on a 4-point scale, from “not at all” (0) to “very much,”^3^ with higher scores indicating a more negative body image. In the present study, the 5 general items were selected in line with previous research on noncancer populations.^4^ The summary score of the BIS was used as predictor in the regression models, single missing items were handled as missing in the summary score. The BIS has shown clinical validity, high test-retest reliability, and good internal consistency in patients with cancer^22^ and has been used in Swedish samples previously.^23^ Cronbach's alpha in the present study was 0.85.

##### Fertility-Related distress

Fertility-related distress was measured using 3 dimensions of the Reproductive Concerns After Cancer (RCAC) scale. The RCAC is a multidimensional scale measuring a range of fertility and parenthood concerns that originally was developed for young adult female cancer survivors.^24^ The RCAC scale includes 18 items measuring 6 dimensions (each dimension consists of 3 items). On each item, responses are given on a 5-point scale (ranging from 1 = strongly disagree to 5 = strongly agree). A high level of distress is defined as a mean score of >4 in the respective dimension. In the present study, the dimensions focusing distress related to having had cancer (partner disclosure, child's health, and personal health) were omitted, leaving the dimensions fertility potential, acceptance, and becoming pregnant. The RCAC scale has demonstrated satisfactory internal consistency and construct validity.^25^ The Swedish version of the scale has been evaluated and shown to have acceptable psychometrical properties.^26^ An individual's subscale mean was calculated provided at least half of subscale items were completed. Cronbach's alpha in the present study ranged from 0.60 to 0.80.

##### Health-Related Quality of Life

Health-related quality of life was assessed using The European Organization for Research and Treatment of Cancer quality of life questionnaire (EORTC QLQ-C30), version 3.0. The QLQ-C30 is a 30-item questionnaire for assessment of the quality of life of patients with cancer^27^^,^^28^ and the general population.^29^ The QLQ-C30 includes 5 functional scales, 3 symptom scales, a global health status scale, and 6 single items. The QLQ-C30 has demonstrated good psychometric properties,^27^^,^^30^ and the Swedish version of the QLQ-C30 has been validated in the general population.^31^ In this study, the summary score was used according to Geisinger et al^28^ and the EORTC QLQ-C30 scoring manual (3rd Edition).^32^ Missing data were handled according to the established guidelines by the EORTC. Cronbach's alpha for the summary score in the present study was 0.77.

### Sociodemographic Variables

Study-specific items were used to assess the age, birth country, education, family situation (children, partner), occupation status, sexual orientation, and wish for (additional) children.

### Statistical Analyses

Outcomes were calculated using descriptive statistics. The chi-square test was used to compare women and men on the demographic variables. The chi-square test was also used to analyze differences between women and men in the number of individuals scoring more than the cutoff for sexual dysfunctions, and t-test was used to calculate differences between women and men in mean scores of the PROMIS SexFS domains. Predictors of sexual dysfunction in all domains of the PROMIS SexFS were analyzed using multivariable logistic regression models, with effects expressed as unstandardized regression coefficients, b. Owing to high correlations between some of the intended predictor variables (Spearman's correlation coefficient >0.5), the QLQ-C30 and the selected RCAC dimensions were not included as x-variables in the models. As the HADS subscales (HADS-A and HADS-D) were highly correlated, we used the HADS-T in the model. The following variables were included as predictors in all models: age (continuous), birth country (other country than Sweden = 1), education (university = 1), having children (yes = 1), occupation (full-time = 1), partnered (yes = 1), wish for (additional) children in the future (yes = 1), BIS (continuous), and HADS-T (continuous).

## Results

### Participants

Of the 2000 individuals who were invited to participate in the study, 59 were excluded because of unknown addresses (n = 41), cognitive impairment (n = 3), language difficulties (n = 2), and/or reporting having been treated for cancer (n = 13). A total of 819 individuals (493 women and 326 men) consented to participate in the study, representing a response rate of 42% (51% for women and 34% for men). Responses were provided via the paper survey by 67% (n = 330) of the women and 51% (n = 160) of the men. Remaining participants gave their responses online.

The mean age of the participants was 29.7 years (SD = 6.1; range 19-40) for women and 29.3 years (SD = 6.4; range 19-40) for men. The demographic characteristics for the sample were comparable with those of the general population in Sweden, besides for the proportion of individuals with another birth country than Sweden;^45^ see Table 1 for participant characteristics and comparison figures for the general population.

### Sexual Function

Among women, 53% reported at least one sexual dysfunction; the corresponding figure for men was 31% (*X*^*2*^ = 41.4, *P* < .001). The most commonly reported sexual dysfunctions in women were low sexual interest (32%) and low orgasm ability (28%), and these were significantly more prevalent among women than men. In men, the most common dysfunction was low satisfaction with sex life (17%), which was significantly more frequently reported by men than by women. There was no significant group difference in the prevalence of dysfunction related to orgasm pleasure. With regard to the overall mean T-score in the domains of the PROMIS SexFS, men reported significantly higher levels of orgasm ability and sexual interest and lower levels of orgasm pleasure than did women. There was no difference between women and men in satisfaction with sex life. For complete results, see Table 2.

### Sexual Activity

A large majority of both women (87%) and men (93%) reported having had sex during the past 30 days (including partner sex and/or masturbation). Of the women, 58% reported having masturbated at least one time during this time period, the corresponding figure in men was 78%. A total of 78% of the women and 62% of the men reported having had sex with a partner during the past 30 days. The most common reason given as to why participants had not had sex with a partner during this period was “too tired” in women and “lack of partner” in men. See Table 3 for sexual activity.

**Table 3:** Sexual activity reported in women and men

	Women n (%)	Men n (%)
Had sexual activity during the past 30 days∗	428 (87)	300 (93)
Had sexual activity with partner during the past 30 days	339 (78)	188 (62)
Reasons for not having had sex with a partner†^,^‡		
Too tired	75 (15)	39 (12)
Not interested in sexual activities	52 (11)	17 (5)
Do not have time/too busy	50 (10)	39 (12)
Felt unattractive	46 (9)	<5
No partner during the past 30 days	45 (9)	58 (18)
Partner did not want to	<5	24 (7)

∗ Including sex with partner and masturbation.

† Frequencies more than 5% are reported in table. In addition, 8% of women and 2% of men reported “other” reason (being pregnant/having a pregnant partner, being sick or having a sick partner, depression and anxiety, waiting till marriage or religious reasons).

‡ Reasons reported according to descending prevalence for women

### Predictors of Sexual Dysfunction

#### Results for Women

For women, having a partner was related to lower risk of dysfunction in the domains Satisfaction with sex life, Sexual interest, and Orgasm pleasure. Being born in another country than Sweden related to a higher risk of vulvar discomfort—clitoral and to a higher risk of dysfunction related to orgasm pleasure. In addition, having children predicted dysfunction related to sexual interest. Finally, anxiety and depression predicted dysfunction related to Satisfaction with sex life, Sexual interest, Vulvar discomfort—Clitoral, and Lubrication. For full model results for women, see Table 4.

**Table 4:** Multivariable logistic binary regression models of cases of sexual dysfunction in the respective PROMIS SexFS domains for women

Type of dysfunction∗^,^†	Satisfaction with sex life	Sexual interest	Orgasm pleasure‡	Vulvar discomfort–clitoral	Lubrication
OR (95% CI)					
Age	0.98 (0.90-1.05)	1.02 (0.98-1.07)	0.98 (0.92-1.05)	0.93§ (0.87-1.00)	0.99 (0.89-1.09)
Having children	1.86 (0.79-4.37)	2.08‖ (1.21-3.59)	0.67 (0.30-1.47)	0.77 (0.35-1.68)	1.10 (0.40-3.04)
Other birth country than Sweden	0.82 (0.28-2.40)	0.99 (0.53-1.84)	2.55§ (1.18-5.51)	2.66§ (1.25-5.68)	2.57 (0.99-6.65)
Partnered	0.26‖ (0.12-0.56)	0.58§ (0.34-0.97)	0.41§ (0.21-0.81)	1.58 (0.70-3.55)	1.69 (0.47-5.99)
HADS-T	1.10¶ (1.05-1.15)	1.05‖ (1.02-1.08)	1.04 (1.00-1.09)	1.07§ (1.03-1.12)	1.06§ (1.00-1.13)

PROMIS SexFS = Patient-Reported Outcomes Measurement Information System Sexual Function and Satisfaction measure, version 2.0; BIS = Body Image Scale; HADS = Hospital Anxiety and Depression Scale.

In addition to the variables reported in the table, the following variables were also controlled for in all models: education (university vs other), occupation (full-time vs other), wish for (additional) children, and the BIS. These variables were all nonsignificant. Regression models for dysfunction with regard to lubrication, orgasm ability, vaginal discomfort, and vulvar discomfort labia showed no significant predictors and are not reported in table.

∗ Answered by individuals who have had sexual activity (with or without partner) during past 30 days.

† PROMIS SexFS cutoff for dysfunction = 1 SD from the T-score mean of the norm population.^49^

‡ Owing to few (<30) cases in the outcome variable, a lower cut-off was used, that is, 0.5 SD.

§ <0.05.

‖ <.01.

¶ <0.001.

#### Results for Men

Model results for men showed that having a partner was related to a lower risk of sexual dysfunction in the domains Satisfaction with sex life and Orgasm pleasure. Having a birth country other than Sweden predicted ED and dysfunction related to Orgasm ability. Symptoms of depression and anxiety was also a significant predictor of dysfunction related to Satisfaction with sex life, Erectile function, and Orgasm pleasure. For complete model results for men, see Table 5.

**Table 5:** Multivariable logistic binary regression models of cases of sexual dysfunction in the respective PROMIS SexFS domains for men

Type of dysfunction∗^,^†	Satisfaction with sex life	Sexual interest	Orgasm ability‡	Orgasm pleasure‡	Erectile function‡
OR (95% CI)					
Have children	4.51§ (1.41-14.39)	0.98 (0.91-1.07)	2.21 (0.47-10.44)	1.73 (0.65-4.60)	0.77 (0.28-2.10)
Other birth country than Sweden	1.06 (0.40-2.82)	1.12 (0.38-3.27)	3.6§ (1.06-12.23)	1.98 (0.82-4.79)	3.0§ (1.27-7.08)
Partnered	0.09¶ (0.03-0.24)	0.46 (0.18-1.20)	1.22 (0.26-5.79)	0.26ǁ (0.12-0.60)	0.62 (0.26-1.47)
Wish for additional children	0.75 (0.36-1.57)	0.34§ (0.14-0.80)	1.62 (0.48-5.48)	0.37ǁ (0.18-0.77)	1.68 (0.76-3.73)
HADS-T	1.09ǁ (1.03-1.16)	1.01 (0.94-1.08)	1.06 (0.97-1.16)	1.16¶ (1.09-1.23)	1.07§ (1.01-1.14)

PROMIS SexFS = Patient-Reported Outcomes Measurement Information System Sexual Function and Satisfaction measure, version 2.0; BIS = Body Image Scale; HADS = Hospital Anxiety and Depression Scale.

In addition to the variables reported in the table, the following variables were also controlled for in all models: age, education (university vs other), occupation (full-time vs other), and the BIS. These variables were all nonsignificant.

∗ Answered by individuals who have had sexual activity (with or without partner) during the past 30 days.

† PROMIS SexFS cutoff for dysfunction = 1 SD from the T-score mean of the norm population.^49^

‡ Owing to few (<30) cases in the outcome variable, lower cut-off was used, that is, 0.5 SD.

§ <0.05.

‖ <.01.

¶ <0.001.

## Discussion

The present results showed that half of young women and one-third of young men report at least one sexual dysfunction. The most frequently reported sexual dysfunction among women was low sexual interest, and among men, low satisfaction with sex life. Lack of partner, having children, being born in another country than Sweden, and reporting symptoms of anxiety and depression were identified as predictors of sexual dysfunction in both women and men. Comparing levels of sexual functioning between genders showed more interest in sexual activities and orgasm ability among men, whereas the level of orgasm pleasure was higher among women. There was no difference between women and men in level of satisfaction with sex life.

Our estimate of at least one sexual dysfunction in men (31%) corresponds to previous results.^35^ The results for prevalence of ED at 4% are in the lower range than previous results,^6^ and our estimate of dysfunction related to low sexual interest at 10% are lower as compared with previous findings (15–25% [4]). The prevalence of orgasm dysfunction (ability and pleasure) in men are in line with the results from others.^8^ In women, our estimate of at least one sexual dysfunction is in the higher range than previous results for women (40–50%, [4]), whereas the prevalence of dysfunction related to sexual interest in our study (32%) and orgasm ability (28%) is slightly lower than previous results for women in this age group in the United States.^2^ Overall, our findings replicate previous results with a tendency toward lower prevalence of sexual dysfunction in both women and men.

Sexual function as indicated by the mean score in the domains of the PROMIS SexFS was higher in men than in women in the domains Sexual interest and Orgasm ability, which is in line with previous research.^46^^,^^47^ However, orgasm pleasure was statistically significantly higher in women than in men, which is a novel finding. Results from a previous publication using the PROMIS SexFS in the United States reported higher sexual function in all domains in men than in women (age 18-44 years),^16^ including orgasm pleasure. The same pattern was also reported in a more recent publication from the United States.^48^ Our results showing a higher level of orgasm pleasure in women thus diverge from previous findings. If our results can be explained by differences in sampling, reporting biases, or cultural differences between the countries, affecting sexual outcomes should be investigated further in future studies.

The present findings on women's sexual activity during the last month are in line with previous results for similar age groups, both regarding the prevalence of any sexual activity (with a partner or alone)^22^ and sexual activity with a partner.^5^ The prevalence of men who reported having been sexually active with a partner was however lower than that reported in a large population-based U.K. study by Mercer et al (62% vs ≈ 75%).^5^ With regard to masturbation, the present findings replicate previous prevalence rates among young men (78%), but show a higher frequency of masturbation among women in Sweden than in the United Kingdom (59% vs 44%).^5^ The same line of reasoning applies to these results as to the results of orgasm pleasure; our findings might reflect a true difference between women in Sweden and the United Kingdom. One can however also speculate whether the results could be explained by a reporting bias implied by women being more accurate and open in Sweden than in the United Kingdom when reporting sexual activities and function. It can also be speculated whether the results for orgasm pleasure and masturbation occurrence are related to each other. It is a possibility that women in Sweden are more satisfied with their orgasms because they also masturbate more frequently or that they masturbate more because they experience their orgasms as more pleasurable. We did however not investigate in this study when the orgasms occurred (with or without a partner) and how satisfactory they were at different occasions. This issue is recommended to be further studied.

The results with regard to predictors of sexual dysfunction showed that immigrants had an increased risk of sexual dysfunction (in the domains Erectile function and Orgasm ability for men and Vulvar discomfort of clitoris and Orgasm pleasure in women). It has previously been reported in a Swedish population that Assyrian/Syrian immigrants with diabetes have a higher risk of also developing sexual dysfunction than native Swedes with diabetes.^49^ Similar results have also been reported in an epidemiological study of Swedish women where first-generation immigrants had a significantly higher prevalence of distressing vaginism.^50^ Our results could be interpreted as an extension of these findings by showing this pattern in both young women and men of the general population. These findings could also be related to research identifying a higher risk of sexual dysfunction in some parts of the world, that is, research reporting a higher prevalence of ED in the United States than in Europe,^7^ and higher levels of sexual problems in women in Asia than in Northern Europe.^36^ Future studies should determine whether our results best can be explained by cultural differences related to the birth country or to the situation of being an immigrant per se.

Predictors of orgasm problems in men have rarely been investigated in previous research. Our study differentiates between problems related to orgasm ability and orgasm pleasure, and results show that predictors of these types of orgasm problems differ. The predictors of dysfunction related to orgasm pleasure in men, symptoms of anxiety and depression, and lack of partner relate to previous studies reporting that having a partner relationship is related to higher sexual satisfaction and women living without a partner experience more orgasm difficulties and physical pain during intercourse.^35^^,^^41^ Dysfunction related to the ability to achieve an orgasm however was not predicted by these factors but instead predicted by having another birth country than Sweden. In women, none of the predictors were significant for orgasm ability, whereas dysfunction related to orgasm pleasure was predicted by having another birth country than Sweden and by a lack of a partner. Our results, showing that the predictors of orgasm pleasure differ from the predictors of orgasm ability, point to possible different mechanisms involved in these aspects of orgasmic dysfunction, which is in line with the previous call for a clarification of the concept of orgasmic disorders.^9^

Finally, poor mental health has been reported to be a main risk factor of sexual dysfunctions in previous research.^37^^,^^12^ Our results validate these findings for young women and men. Interestingly, a negative body image did not predict sexual dysfunction, whereas symptoms of anxiety and depression were related to several types of sexual dysfunction in both women and men. These results underscore the importance of assessing depression and anxiety in clinical care of sexual dysfunctions, as well as considering the increased risk of sexual dysfunctions in young adult patients with depression and anxiety.

### Limitations and Strengths

A strength of the present study is the use of a population-based data collection method. Comparison with public statistics for young adults in the general Swedish population indicates that our sample is representative in terms of key demographic variables, except with regard to the birth country. Study participants seem to include a smaller proportion of immigrants than the total population, which is not surprising because our survey was only available in Swedish, but should still be considered when interpreting the results. In the present study, we used a self-assessment scale to determine prevalence of sexual dysfunction. It is worth to notice that the definition of “sexual dysfunction” used in our study is more in line with epidemiological studies assessing prevalence of a condition in a population than studies using clinical definitions where duration, severity, and bother may need to be part of the evaluation.^1^ Because we did not include bother or duration of problems in our definition of sexual dysfunction, the prevalence estimates may thus imply an overestimation of sexual dysfunction of practical and clinical relevance. Still, assessing the level of problems experienced by the participants is in line with the method used in previous studies with similar aims.^6^ A strength of the measurement scale used in the present study, the SexFS, is that it includes self-report of sexual activities performed with or without a partner regardless of sexual orientation. This allows investigation of sexual problems in a large proportion of the population and identification of problems occurring in all types of sexual activities. Several previous measures of sexual function have been critiqued for being suitable mainly for administration to people in a current, and often heterosexual, relationship.^51^ A possible limitation of this study is the use of U.S. norms to interpret the SexFS, as these might not correspond sufficiently well to the Swedish population. In addition, the norms for the SexFS are for a broader age span than used in this study, which may hamper the comparison. Still, many aspects are similar between the United States and Sweden including the age for sexual debut.^52^ Swedish norm data and norms for younger adults specifically for the SexFS should however be collected to further improve the quality of comparisons using this instrument in the future. The low Cronbach's alpha score for the ED domain of the SexFS in the present study should also be acknowledged. This may be explained by the men in the present study having a high level of erectile function with approximately 85% ceiling effects on each of the 3 items. This domain was developed and initially psychometrically evaluated in a sample also including older men with a larger proportion of dysfunction and was shown to have satisfactory internal consistency.^15^ Another possible limitation of the present study is the lack of assessment of medical comorbidities as conditions such as heart disease or diabetes have been reported to be associated to sexual dysfunction in women and men up to the age of 74 years.^53^ We did assess health-related quality of life, which partly can capture medical disease burden, but removed this variable from the regression analyses because of high correlation with symptoms of anxiety and depression. This choice was made as sociodemographic and psychological variables can be considered as more important in a young population where medical conditions are less prevalent.^54^ Still, having assessed medication such as antidepressants would have been beneficial to draw conclusion with regard to mechanisms involved. Another potential limitation to consider is the response rate of 42%, which implies a risk bias in the estimates. Although this is a higher response rate than reported in other studies investigating sexual function in the general population (eg, see the study by Giesinger et al^28^), the risk of selection bias should still be taken into consideration when interpreting the results. It should also be acknowledged that the variables in the models were assessed simultaneously, which hampers conclusions regarding causal relationships. Future studies using longitudinal designs are encouraged to validate relationships between, for example, anxiety and depression and sexual dysfunction. Finally, as we performed 5 model analyses for women and 5 for men, some caution with regard to multiple comparisons and inflation errors should be made. If we would have used a more strict level to detect a significant predictor variable, some of the variables included in the models would have turned out nonsignificant. For women, the predictor variables for the outcomes Orgasm pleasure, Vulvar discomfort–Clitoral, and Lubrication were significant at the 0.05 level and should be interpreted with specific caution. Correspondingly, the predictor variables for Orgasm ability and Erectile function were significant on the 0.05 level for men. These results could have to do with the sample size being too small for the prevalence of cases in the outcome variable or that the predictor was significant because of a type 1 error. Future larger studies should investigate these relationships further.

## Conclusions

Our results, using validated measurements of sexual dysfunction, show that half of young women and one-third of young men report at least one sexual dysfunction. Overall, men report more sexual interest and greater ability to achieve orgasm than women. The most frequently reported sexual dysfunction among women was low sexual interest and among men low satisfaction with sex life. Our results also showed that immigrants have a higher risk of sexual dysfunction, which should be investigated in upcoming studies to determine mechanisms involved. Finally, our findings underscore that symptoms of anxiety and depression are important predictors of sexual dysfunction in both young women and men, which should be acknowledged by clinicians.

## Statement of authorship

Lisa Ljungman: Conceptualization, Methodology, Formal Analysis, Writing - Original draft, Writing - Review & Editing; Claudia Lampic: Conceptualization, Methodology, Formal Analysis, Investigation, Writing - Original draft, Writing - Review & Editing, Funding Acquisition; Lena Wettergren: Conceptualization, Methodology, Formal Analysis, Investigation, Writing - Original draft, Writing - Review & Editing, Funding Acquisition.

## References

[1] Lewis R.W., Fugl-Meyer K.S., Corona G. (2010). Definitions/epidemiology/risk factors for sexual dysfunction. J Sex Med.

[2] Shifren J.L., Monz B.U., Russo P.A. (2008). Sexual problems and distress in United States women: prevalence and correlates. Obstet Gynecol.

[3] Lau J.T.F., Kim J.H., Tsui H.Y. (2005). Prevalence of male and female sexual problems, perceptions related to sex and association with quality of life in a Chinese population: a population-based study. Int J Impot Res.

[4] Lammerink E.A.G., de Bock G.H., Pascal A. (2017). A survey of female sexual functioning in the general Dutch population. J Sex Med.

[5] Mercer C.H., Tanton C., Prah P. (2013). Changes in sexual attitudes and lifestyles in Britain through the life course and over time: findings from the national surveys of sexual attitudes and Lifestyles (Natsal). Lancet [Internet].

[6] McCabe M.P., Sharlip I.D., Lewis R. (2016). Incidence and prevalence of sexual dysfunction in women and men: a Consensus Statement from the Fourth international Consultation on sexual medicine 2015. J Sex Med [Internet].

[7] Rosen R.O., Fisher W.A., Eardley I. (2004). The multinational Men’s Attitudes to Life Events and Sexuality (MALES) study: I. Prevalence of erectile dysfunction and related health concerns in the general population. Curr Med Res Opin.

[8] Laumann E.O., Paik A., Rosen R.C. (1999). Sexual dysfunction in the United States. J Am Med Assoc.

[9] Waldinger M.D., Schweitzer D.H. (2005). Retarded ejaculation in men: an overview of psychological and neurobiological insights. World J Urol.

[10] Smith A.M.A., Lyons A., Ferris J.A. (2013). Incidence and persistence/recurrence of men’s sexual difficulties: findings from the Australian longitudinal study of health and relationships. J Sex Marital Ther.

[11] Rosen R.C. (2000). Prevalence and risk factors of sexual dysfunction in men and women. Curr Psychiatry Rep.

[12] Nguyen H.M.T., Gabrielson A.T., Hellstrom W.J.G. (2017). Erectile dysfunction in young men—a review of the prevalence and risk factors. Sex Med Rev [Internet].

[13] Ponholzer A., Temml C., Mock K. (2005). Prevalence and risk factors for erectile dysfunction in 2869 men using a validated questionnaire. Eur Urol.

[14] Rastrelli G., Maggi M. (2017). Erectile dysfunction in fit and healthy young men: psychological or pathological?. Transl Androl Urol.

[15] Lampic C., Ljungman L., Micaux Obol C. (2019). A web-based psycho-educational intervention (Fex-Can) targeting sexual dysfunction and fertility-related distress in young adults with cancer: study protocol of a randomized controlled trial. BMC Cancer.

[16] Weinfurt K.P., Lin L., Bruner D.W. (2015). Development and initial validation of the PROMIS® sexual function and satisfaction measures version 2.0. J Sex Med [Internet].

[17] Strandquist J., Eriksson L., Winterling J. (2016). Translation of selected items of the PROMIS SexFS v2.0 into Swedish. The Second PHO Conference, October 23-24. Copenhagen.

[18] Zigmond A.S.S.R. (2008). The hospital anxiety and depression scale. Acta Psychiatr Scand.

[19] Singer S., Kuhnt S., Götze H. (2009). Hospital anxiety and depression scale cutoff scores for cancer patients in acute care. Br J Cancer.

[20] Bjelland I., Dahl A.A., Tangen T. (2002). The validity of the Hospital Anxiety and Depression Scale: an updated literature review. J Psychosom Res.

[21] Lisspers J., Nygren A., Söderman E. (1997). Hospital anxiety and depression scale (HAD): some psychometric data for a Swedish sample. Acta Psychiatr Scand.

[22] Hopwood P., Fletcher I., Lee A. (2001). A body image scale for use with cancer patients. Eur J Cancer [Internet].

[23] Brandberg Y., Sandelin K., Erikson S. (2008). Psychological reactions, quality of life, and body image after bilateral prophylactic mastectomy in women at high risk for breast cancer: a prospective 1-year follow-up study. J Clin Oncol.

[24] Gorman J.R., Su H.I., Pierce J.P. (2014). A multidimensional scale to measure the reproductive concerns of young adult female cancer survivors. J Cancer Surviv [Internet].

[25] Gorman J.R., Su H.I., Roberts S.C. (2015). Experiencing reproductive concerns as a female cancer survivor is associated with depression. Cancer.

[26] Anandavadivelan P., Wiklander M., Eriksson L.E. (2020). Cultural adaptation and psychometric evaluation of the Swedish version of the Reproductive Concerns After Cancer (RCAC) scale. Health Qual Life Outcomes.

[27] Aaronson N.K., Ahmedzai S., Bergman B. (1993). The european organisation for research and treatment of cancer QLQ-C30: a quality-of-life instrument for use in international clinical trials in oncology. J Natl Cancer Inst.

[28] Giesinger J.M., Kieffer J.M., Fayers P.M. (2016). Replication and validation of higher order models demonstrated that a summary score for the EORTC QLQ-C30 is robust. J Clin Epidemiol [Internet].

[29] Michelson H., Bolund C., Nilsson B. (2000). Health-Related Quality of Life measured by the EORTC QLQ-C30 -Reference values from a large sample of the Swedish population. Acta Oncol (Madr).

[30] Shih C.L., Chen C.H., Sheu C.F. (2013). Validating and improving the reliability of the EORTC QLQ-C30 using a multidimensional rasch model. Value Heal [Internet].

[31] Derogar M., Van Der Schaaf M., Lagergren P. (2012). Reference values for the EORTC QLQ-C30 quality of life questionnaire in a random sample of the Swedish population. Acta Oncol (Madr).

[32] EORTC Data Center (2001). EORTC QLQ-C30 scoring Manual the EORTC QLQ-C30 introduction. EORTC Qlq-c30 Scoring Man EORTC Qlq-c30 Introd [Internet].

[33] Richters J., Grulich A.E., De Visser R.O. (2003). Attitudes towards sex in a representative sample of adults. Aust N Z J Public Health.

[34] Hayes R.D., Bennett C.M., Fairley C.K. (2006). What can prevalence studies tell us about female sexual difficulty and dysfunction?. J Sex Med.

[35] Smith A.M.A., Lyons A., Ferris J.A. (2012). Incidence and persistence/recurrence of women’s sexual difficulties: findings from the Australian longitudinal study of health and relationships. J Sex Marital Ther.

[36] Nicolosi A., Laumann E.O., Glasser D.B. (2004). Sexual behavior and sexual dysfunctions after age 40: the global study of sexual attitudes and behaviors. Urology.

[37] Shahhosseini Z., Gardeshi Z., Pourasghar M. (2014). A review of affecting factors on sexual satisfaction in women. Mater Socio Med.

[38] Quinn-Nilas C., Benson L., Milhausen R.R. (2016). The relationship between body image and domains of sexual functioning among heterosexual, Emerging adult women. Sex Med [Internet].

[39] Facchin F., Somigliana E., Busnelli A. (2019). Infertility-related distress and female sexual function during assisted. Hum Reprod.

[40] Yoo H., Bartle-Haring S., Day R.D. (2014). Couple communication, emotional and sexual intimacy, and relationship satisfaction. J Sex Marital Ther.

[41] Pedersen W., Blekesaune M. (2003). Sexual satisfaction in young adulthood: Cohabitation, committed dating or unattached life?. Acta Sociol.

[42] Ljungman L., Ahlgren J., Petersson L. (2018). Sexual dysfunction and reproductive concerns in young women with breast cancer: type, prevalence, and predictors of problems. Psychooncology.

[43] Coskun B., Coskun B.N., Atis G. (2013). Evaluation of sexual function in women with rheumatoid arthritis. Urol J.

[44] McCabe M.P., Sharlip I.D., Lewis R. (2016). Incidence and prevalence of sexual dysfunction in women and men: A consensus statement from the Fourth International Consultation on Sexual Medicine 2015. J Sex Med.

[45] Statistics Sweden, SCB [Internet]. https://www.scb.se/om-scb/samordning-av-sveriges-officiella-statistik/.

[46] Flynn K.E., Lindau S.T., Lin L. (2015). Development and validation of a single-item screener for self-reporting sexual problems in U.S. adults. J Gen Intern Med.

[47] Folkhälsomyndigheten. Sexuell och reproduktiv hälsa och rättigheter i Sverige 2017. 2019. Available at: folkhalsomyndigheten.se/publicerat-material/publikationsarkiv/s/sexuell-och-reproduktiv-halsa-och-rattigheter-i-sverige-2017/. Accessed September 22, 2020.

[48] Flynn K.E., Lin L., Weinfurt K.P. (2017). Sexual function and satisfaction among heterosexual and sexual minority U.S. Adults: a cross-sectional survey. PLoS One.

[49] Taloyan M., Wajngot A., Johansson S.E. (2012). Sexual dysfunction in Assyrian/Syrian immigrants and Swedish-born persons with type 2 diabetes. BMC Res Notes [Internet].

[50] Öberg K., Fugl-Meyer A.R., Fugl-Meyer K.S. (2004). On categorization and quantification of women’s sexual dysfunctions: an epidemiological approach. Int J Impot Res.

[51] Daker-White G. (2002). Reliable and valid self-report outcome measures in sexual (dys)function: a systematic review. Arch Sex Behav.

[52] Danielsson M., Berglund T., Forsberg M. (2012). Health in Sweden: the national public health report 2012. Chapter 9. Scand J Public Health.

[53] Polland A., Davis M., Zeymo A. (2018). Comparison of correlated comorbidities in male and female sexual dysfunction: findings from the third national survey of sexual attitudes and lifestyles (Natsal-3). J Sex Med [Internet].

[54] Egunsola O., Raubenheimer J., Buckley N. (2019). Variability in the burden of disease estimates with or without age weighting and discounting: a methodological study. BMJ Open.

